# Healthy Hospitals

**Published:** 1894-06

**Authors:** 


*Healthy Hospitals.* By Sir DOUGLAS GALTON. Pp. xi., 287. Oxford: At the Clarendon Press. 1893.

This work does not embody the detailed requirements of hospitals for special diseases, but is limited to explaining the general principles upon which the healthy construction of all hospitals must be based. In this endeavour the author has been, on the whole, successful, though some points receive inadequate treatment; as, for instance, the "drainage" of the ward-unit, a point of no small importance, which is summarily dismissed in two pages. The chapters on "Ventilation" and "Warming" are, on the other hand, very full, and include descriptions of the latest developments of mechanical ventilation. The remarks on the comparative value of ventilation by mechanical and natural means, and on the advantages of simplicity in this as in other points of hospital design, are judicious and well stated. We miss, in the account of methods proposed for the cremation of outgoing air from infectious disease wards, any mention of the failure of this method at the Bradford Smallpox Hospital, an omission of some importance. As a whole, the work supplies trustworthy and useful information upon a variety of points essential to the construction of healthy hospitals.

